# Fully Automated Azeotropic Drying-Free Synthesis of [^18^F]SynVesT-1 via Copper-Mediated Fluorination Using the Trasis All-in-One Module

**DOI:** 10.3390/molecules31132396

**Published:** 2026-07-07

**Authors:** Falguni Basuli, Jianfeng Shi, Xiang Zhang, Rolf E. Swenson

**Affiliations:** Chemistry and Synthesis Center, National Heart, Lung, and Blood Institute, National Institutes of Health, Bethesda, MD 20892, USA

**Keywords:** fluorine-18, 4-dimethylaminopyridine (DMAP), copper-mediated, labeling, automated, Sep-Pak, Trasis All-in-One, PET

## Abstract

Synaptic vesicle glycoprotein 2A (SV2A) is a 12-pass transmembrane protein expressed in presynaptic vesicles. Positron emission tomography (PET) imaging of SV2A provides an in vivo measure of synaptic density and has broad applications in the study of neuropsychiatric and neurodegenerative diseases. A fluorine-18-labeled PET tracer targeting SV2A, [^18^F]SynVesT-1, was originally developed using a copper-mediated fluorination approach that requires initial azeotropic drying of [^18^F]fluoride with anhydrous acetonitrile. We previously established an efficient radiolabeling strategy in which fluorine-18 retained on an anion-exchange cartridge is eluted as 4-dimethylaminopyridinium [^18^F]fluoride (DMAPH·[^18^F]F) by passing a solution of 4-dimethylaminopyridinium trifluoromethanesulfonate (DMAPH·OTf) in dimethylacetamide (DMA), which was directly used in copper-mediated fluorination of various substrates without the need for azeotropic drying. Building on this strategy, we developed a fully automated and reproducible method for the synthesis of [^18^F]SynVesT-1 using the Trasis All-in-One (AIO) module. The total synthesis time was 60 min, affording a superior overall decay-corrected radiochemical yield (30–37% vs. 20.6 ± 1.2%) while requiring a reduced amount of precursor (2 mg vs. 5 mg) with a radiochemical purity greater than 98%.

## 1. Introduction

Synaptic vesicle glycoprotein 2A (SV2A) is the most widely distributed synaptic vesicle transmembrane protein, present in nearly all synaptic terminals regardless of neurotransmitter type [1,2,3]. SV2A plays a crucial role in neurotransmitter release and the maintenance of synaptic vesicle homeostasis. Due to its ubiquitous expression throughout gray matter regions, SV2A has emerged as a promising marker of synaptic density, motivating the development of molecules derived from the anti-epileptic drug levetiracetam [(S)-α-ethyl-2-oxo-pyrrolidine acetamide], commercially known as Keppra^®^ [4,5,6]. Alterations in synaptic structure are linked to a wide range of neurodegenerative disorders, including Alzheimer’s disease (AD), epilepsy, Parkinson’s disease (PD), and autism spectrum disorders, as well as psychiatric conditions such as depression and schizophrenia [7,8,9,10,11,12,13,14,15,16,17,18,19]. Beyond the central nervous system, SV2A is also expressed in neuroblastoma and in neuroendocrine tissues, including the pancreas, pituitary, and adrenal medulla, and it has been proposed as a marker of neuroendocrine differentiation in neuroendocrine tumors [20,21,22]. Consequently, SV2A represents an attractive target for non-invasive diagnostic imaging. In vivo PET imaging of SV2A therefore provides a quantitative measure of synaptic density and has emerged as a valuable biomarker for a wide range of neurological and psychiatric disorders [23,24,25,26].

In 2016, Nabulsi et al. developed (R)-1-((3-[^11^C]methyl-pyridin-4-yl)methyl)-4-(3,4,5-trifluorophenyl)pyrrolidin-2-one, known as [^11^C]UCB-J, and demonstrated its suitability as a PET tracer for quantifying SV2A protein in nonhuman primates and humans [27,28,29]. However, among the many available PET radionuclides (e.g., carbon-11, fluorine-18, copper-64, gallium-68, and zirconium-89), fluorine-18 is the most commonly used positron emitter for routine imaging due to its favorable properties, such as ease of production and an optimal half-life (t_1/2_ = 110 min), which supports efficient synthesis, transportation, and imaging workflows [30,31,32,33,34,35,36,37]. In contrast, the relatively short half-life helps minimize the patient’s overall radiation exposure. Moreover, due to the low positron energy (634 keV; β^+^, 97%), fluorine-18 decay results in a short positron range in tissue, leading to high-resolution PET images [38]. Therefore, the same group developed a fluorine-18-labeled analog of UCB-J, [^18^F]SynVesT-1 ((R)-4-(3-fluoro-5-(fluoro-^18^F)phenyl)-1-((3-methylpyridin-4-yl)methyl)pyrrolidin-2-one, also known as [^18^F]SDM-8 or [^18^F]MNI-1126), and showed that this tracer retains the favorable characteristics of [^11^C]UCB-J, including high brain uptake and robust specific binding [39].

Conventional aromatic nucleophilic fluorination to form an ^18^F-C bond typically relies on the activation provided by an electron-withdrawing group located ortho or para to the leaving group [33,40]. Since [^18^F]SynVestT-1 lacks such a substitution, its synthesis must proceed via one of the recently developed alternative radio-fluorination strategies, such as those involving iodonium, sulfonium, boronate, or stannane precursors [40,41,42,43,44,45,46]. Li et al. first explored fluorine-18 labeling via an iodonium ylide precursor; however, this approach yielded a very low radiochemical yield (<1%, decay-corrected) and limited specific activity (25.6 MBq/nmol). In contrast, subsequent copper-mediated radiofluorination strategies using boronic ester or trimethyltin precursors produced markedly improved yields. Among these, the trimethyltin precursor afforded the highest radiochemical yield (19%, decay-uncorrected) and specific activity (241.7 MBq/nmol), with a synthesis time of approximately 95 min, highlighting its relative suitability for efficient fluorine-18 labeling to prepare [^18^F]SynVestT-1 [39]. Dahl et al. reported a fully automated radiosynthesis using a commercially available module (TracerMaker, ScanSys Laboratorieteknik ApS, Copenhagen, Denmark) [47]. Recently, Chen et al. successfully translated the procedure to the more versatile Trasis All-in-One (AIO) module with minor adjustments [48]. The Trasis AIO module offers a flexible, fully automated solution for radiopharmaceutical synthesis by supporting diverse radiolabeling chemistries, customizable workflows, integrated HPLC purification, and interchangeable cassette configurations. These capabilities make it well suited for both tracer development and the routine production of PET radiopharmaceuticals. However, both studies used the conventional fluorine-18 catch-and-release strategy followed by azeotropic drying to generate activated [^18^F]fluoride, which is time-consuming and may limit overall synthesis efficiency [47,48]. Our group recently developed a simple fluorination method without azeotropic drying of [^18^F]fluoride. In this approach, [^18^F]F^−^ was eluted as 4-dimethylaminopyridinium [^18^F]fluoride (DMAPH·[^18^F]F) in an anhydrous solvent as used directly for fluorine-18 labeling [49]. Building upon this approach, a fully automated synthesis of [^18^F]SynVestT-1 has now been established on a cassette-based Trasis All-in-One (AIO) module. The simplicity of this method will allow for large-scale routine production of the tracer in a current good manufacturing practice (cGMP) laboratory.

## 2. Results and Discussion

**Scheme 1:**
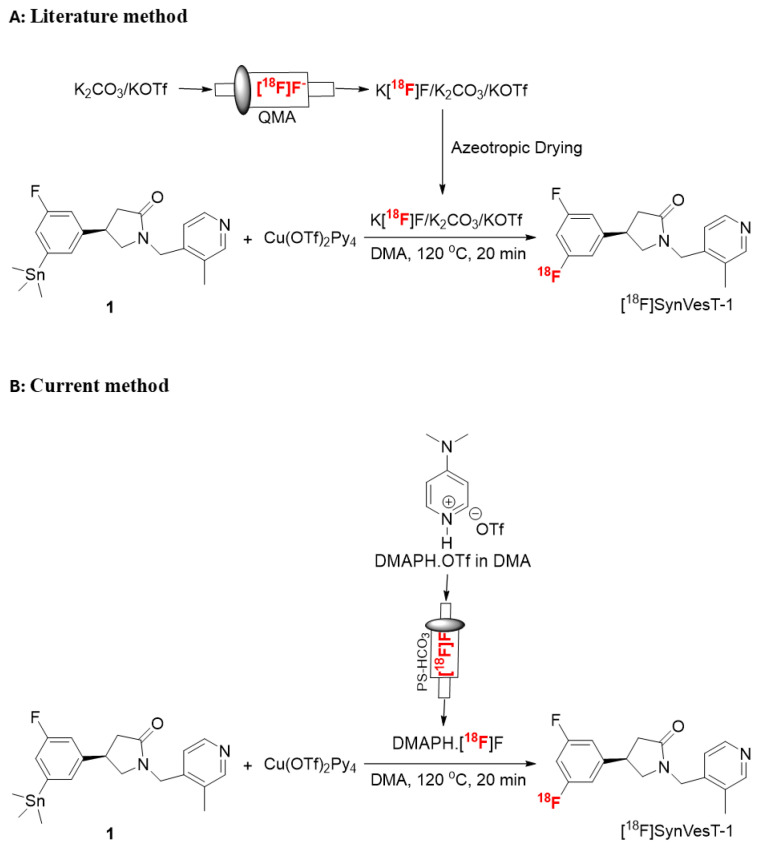
Synthesis of [^18^F] SynVesT-1 [48].

Li et al. first reported the manual synthesis of [^18^F]SynVesT-1 via copper-mediated fluorination with a high radiochemical yield (RCY) [39]. For in-depth clinical investigation, however, an automated, reproducible, and safe multidose production process that complies with good manufacturing practice (GMP) is desirable. To meet these requirements, Dahl et al. developed a fully automated radiosynthesis of [^18^F]SynVesT-1 [47]. With only minor protocol modifications, the method achieved high radiochemical yield while satisfying established acceptance criteria and quality control standards. More recently, Chen et al. (2024) reported an automated synthesis of [^18^F]SynVesT-1 (Scheme 1A) on the widely adopted and more versatile Trasis All-in-One (AIO) module with further minor modifications [48]. Both automated approaches involve a conventional fluorine-18 labeling workflow (Table 1), in which [^18^F]fluoride in target water is retained on an anion-exchange (QMA) cartridge, eluted using a potassium carbonate (K_2_CO_3_) and potassium trifluoromethanesulfonate (KOTf) mixture, and subsequently dried azeotropically with anhydrous acetonitrile to generate reactive K [^18^F]F. The trimethyltin precursor **1** (Scheme 1), in the presence of [Cu(OTf)_2_(py)_4_], was reacted with dried K [^18^F]F at 120 °C for 20 min, followed by HPLC purification. The final formulation of the product was performed using a Sep-Pak tC18 Plus short cartridge catch-and-release to obtain pure [^18^F]SynVesT-1. While robust and well-established, this multistep processing of [^18^F]fluoride remains time-consuming and may destabilize base-sensitive precursors, thereby affecting overall synthesis efficiency.

**Figure 1:**
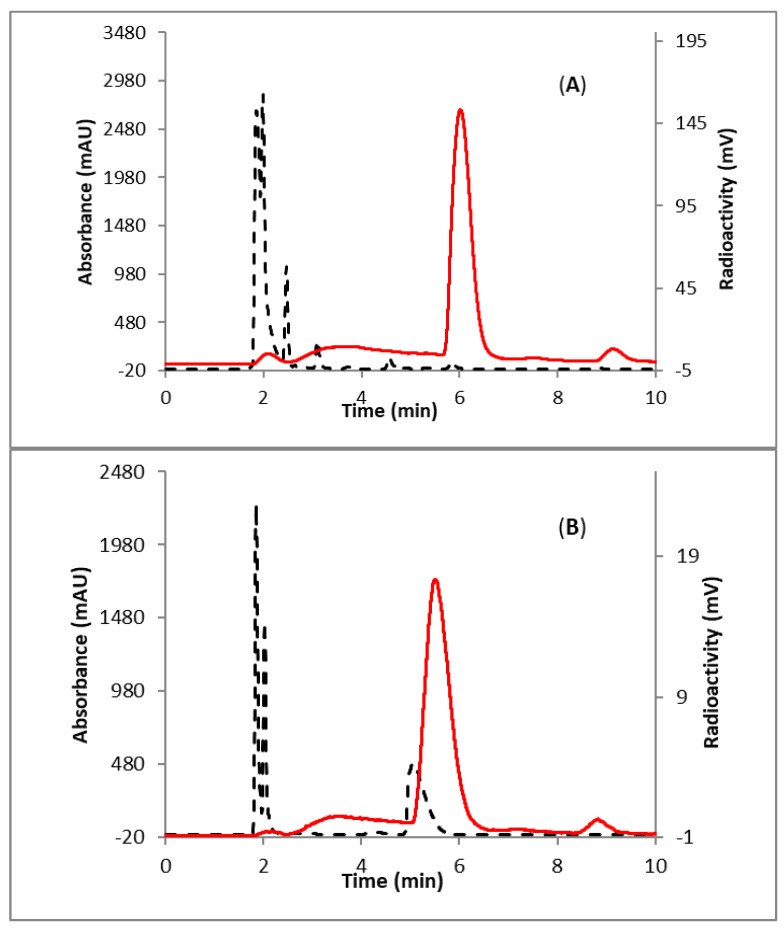
HPLC chromatogram of [^18^F]SynVesT-1; (**A**) reaction mixture; (**B**) reaction mixture co-injected with non-radioactive standard. Black line UV detector at 254 nm, red line radio detector.

In 2017, our group developed a simplified copper-mediated fluorination method designed to streamline the initial processing of fluorine-18 [49]. This approach involves trapping fluorine-18 on a PS-HCO_3_ cartridge (10 mg), followed by drying the cartridge via flushing with anhydrous acetonitrile and under vacuum. The retained fluorine-18 was then eluted with DMAPH·OTf in anhydrous dimethylacetamide (DMA) and used directly for radiolabeling a variety of molecules without azeotropic drying. The elution efficiency was >95%. This method eliminates the need for a base to elute [^18^F]fluoride from the Sep-Pak cartridge and avoids azeotropic drying. As a result, it is well-suited for the radiolabeling of base-sensitive precursors and tracers. Furthermore, the copper reagent will be more stable under these conditions [50,51]. Accordingly, this approach was employed to develop an improved radiolabeling strategy for the synthesis of [^18^F]SynVestT-1 (Scheme 1 and Table 1). The procedure was initially optimized manually using 2 mg of trimethyltin precursor and 15 mg of Cu(OTf)_2_Py_4_. Analysis of the crude reaction mixture by analytical HPLC revealed a major radioactive peak at 6 min (Figure 1A). Co-injection with the corresponding non-radioactive standard confirmed the identity of [^18^F]SynVest (Figure 1B). Unlike the literature method, the reaction mixture was diluted with water (5 mL) and directly injected into the HPLC to produce pure [^18^F]SynVestT-1. The HPLC eluent was also changed to the directly injectable buffer (20% ethanol in 50 mM phosphoric acid), so there was no need for a final Sep-Pak catch-and-release. This approach eliminates several additional processing steps, including collection of the HPLC fraction in a separate vial, trapping of the product on a preconditioned Sep-Pak cartridge, washing with water, and elution with ethanol, thereby reducing overall processing time.

The effect of precursor amount (1–5 mg) on radiochemical yield (RCY) was investigated using 15 mg of Cu(OTf)_2_Py_4_ (Table 2). A high RCY was obtained with 5 mg of precursor (entry 1). This method provided higher RCY than the literature procedure based on azeotropic drying of [^18^F]fluoride (30–37% vs. 20.6 ± 1.2%) [48]. Reducing the precursor amount to 2 mg resulted in only a slight decrease in RCY (entry 2). In contrast, a substantial decrease in RCY was observed at the lowest precursor amount (1 mg). The RCY of this method is higher than that of the literature method, which involves azeotropic drying of the [^18^F]fluoride. The influence of the amount of Cu(OTf)_2_Py_4_ on RCY was also examined (entries 5–8). RCY increased with increasing amounts of Cu(OTf)_2_Py_4_, reaching a maximum at 9 mg (entry 5), corresponding to a 1:6 precursor-to-Cu(OTf)_2_Py_4_ ratio. Further increasing the amount of Cu(OTf)_2_Py_4_ to 15 mg did not improve the radiochemical yield. Elution of [^18^F]fluoride from the PSHCO_3_ cartridge (10 mg) using Cu(OTf)_2_Py_4_ (entry 9) was evaluated according to the reported literature procedure [52]. The elution efficiency was higher than that previously reported (68% vs. 31.6 ± 9.1%), potentially due to the smaller cartridge employed in this study (10 mg vs. 45 mg). However, the RCYfor the synthesis of [^18^F]SynVesT-1 was substantially lower than that obtained using DMAPH·OTf as the eluent (<2% vs. 30–37%). Additionally, changing the radiolabeling solvent to dimethylformamide (DMF, entry 10) resulted in a significant decrease in RCY (Table 2). Lowering the reaction temperature to 110 °C resulted in a reduced RCY (entry 6). The overall RCY of the reaction was 40% (decay-corrected) in 60 min synthesis time using 2 mg of precursor.

**Figure 2:**
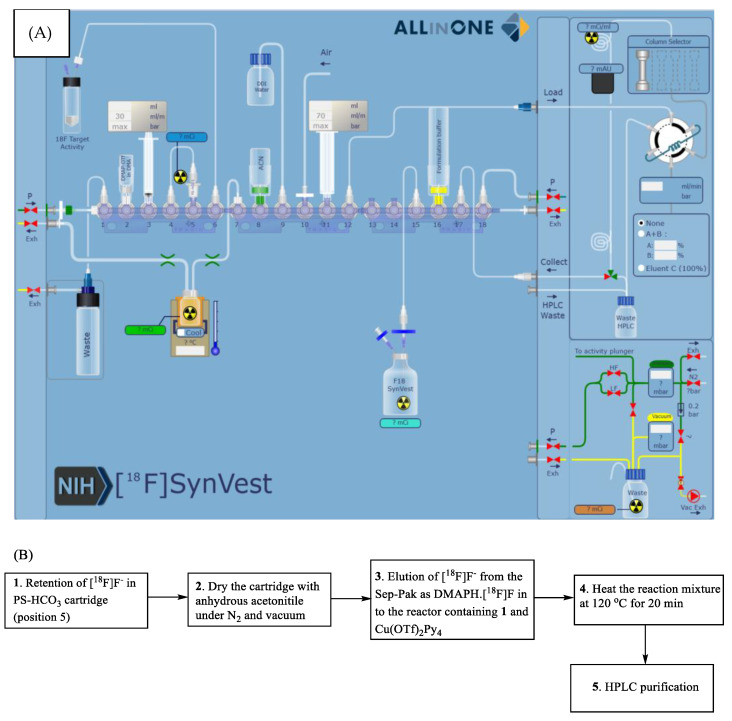
An automated synthesis of [^18^F]SynVest. (**A**) Program layout of Trasis AIO module; (**B**) key steps.

**Figure 3:**
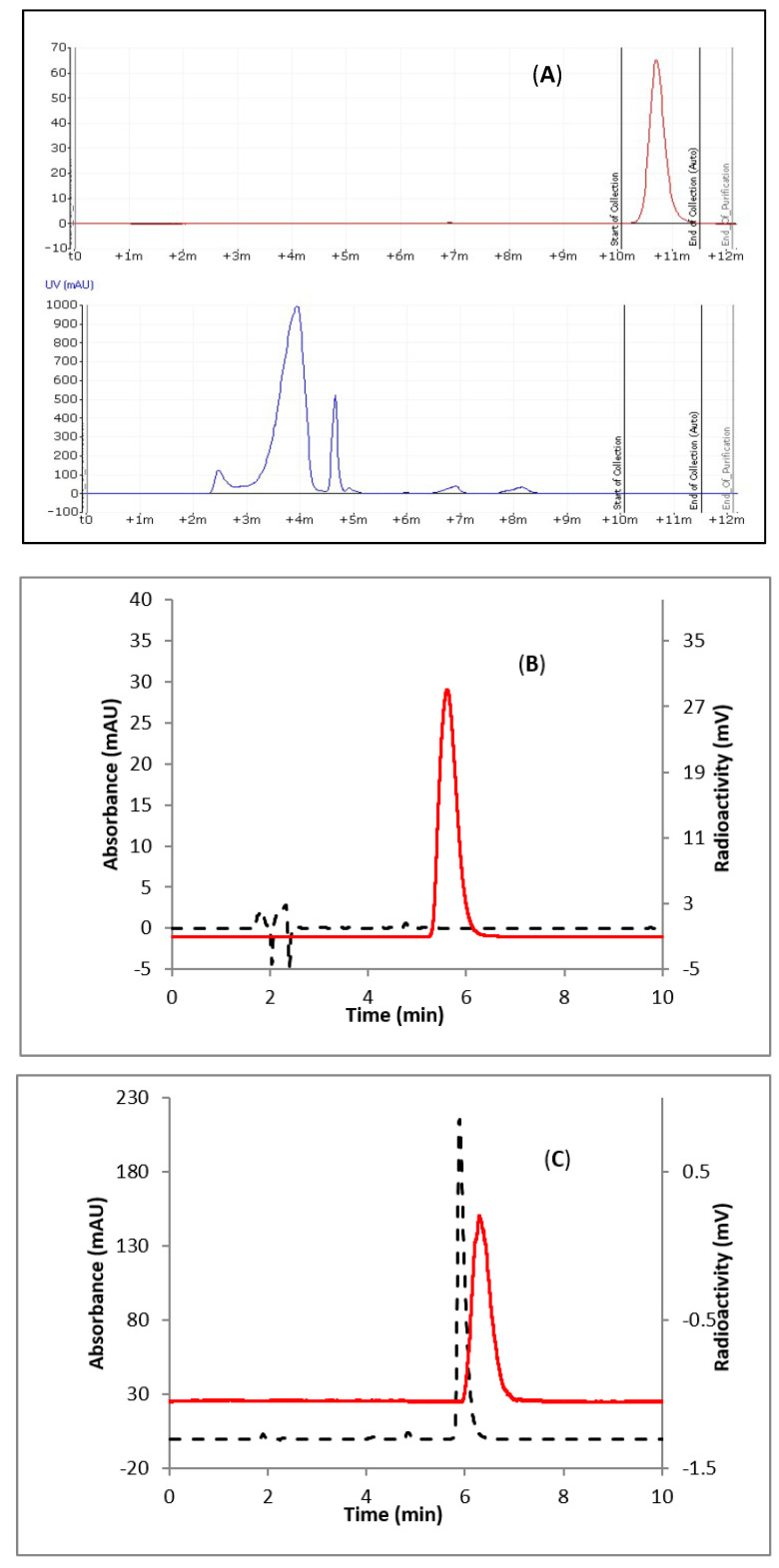
A representative HPLC chromatogram of [^18^F]SynVest; (**A**) semi-prep for purification; (**B**) purified product; (**C**) co-injected with non-radioactive standard. Blue (**A**) or black line (**B**,**C**) UV detector at 254 nm, red line radio detector.

Following successful optimization of the manual procedure, automated synthesis was performed. Due to its operational flexibility, the AIO module was selected to develop a fully automated, cassette-based production of [^18^F]SynVestT-1. A schematic diagram of the automated setup and key synthesis steps is shown in Figure 2. A representative semi-preparative HPLC chromatogram is shown in Figure 3A. The automated synthesis afforded an overall decay-corrected RCY of 30–37% (*n* = 6) with a total synthesis time of 60 min and a radiochemical purity exceeding 98% (Figure 3B). The identity of the final tracer was confirmed by HPLC co-elution with a non-radioactive standard (Figure 3C). The molar activities of [^18^F]SynVestT-1 were 14,000–17,000 Ci/mmol (518–629 GBq/mmol, *n* = 6). The enantiomeric purity (>99%) was measured using a chiral analytical column (LUX^®^, Cellulose-1, 250 mm × 4.6 mm, 5 µm). In representative radiolabeling, starting with 154 mCi (5.7 GBq), 39 mCi (1.4 GBq) of [^18^F]SynVestT-1 was obtained in 60 min. Table 3 summarizes the activities measured at different stages of a representative radiolabeling run performed on the Trasis AIO system. The reported values represent approximate estimates, as the measurements may be influenced by background radioactivity.

To assess the reproducibility of the method, the labeling was performed multiple times (*n* = 6) using 2 mg of precursor **1**. The results for three representative batches are summarized in Table 4.

## 3. Materials and Methods

Trimethyltin precursor and non-radioactive standard were purchased from ABX (Radeberg, Germany). All other chemicals and solvents were received from Sigma-Aldrich (St. Louis, MO, USA) and used without further purification. Fluorine-18 was obtained from the National Institutes of Health cyclotron facility (Bethesda, MD, USA). 10 mg PS-HCO_3_ anion-exchange cartridges were purchased from Synthra (Hamburg, Germany). High-performance liquid chromatography (HPLC) columns used in this synthesis were obtained from Agilent Technologies (Santa Clara, CA, USA). The semi-prep HPLC purification and analytical HPLC analyses for radiochemical work were performed on an Agilent 1200 Series instrument from Agilent Technologies (Santa Clara, CA, USA) equipped with multi-wavelength detectors. The automated radiosynthesis was performed on the Trasis AIO synthesizer from Trasis (Liège, Belgium). Single-use cassettes for the preparation of [^18^F]SynVestT-1 were assembled in-house using Trasis manifolds and components. The residual amount of copper and tin in the final dose was determined by Robertson Microlit Laboratories (Ledgewood, NJ, USA) using ICP-MS.

### 3.1. HPLC Method

#### 3.1.1. Semi-Prep Method for Purification

Column: Phenomenex Luna C-18 (2), 5 µm, 100 A, 250 mm × 10 mm; Eluent: 20% ethanol in 50 mM o-phosphoric acid; Flow rate 4 mL/min, t*_R_* = ~11 min. 

#### 3.1.2. Analytical Method for Quality Control (QC)

Column: Agilent, Eclipse, plus, C-18, 150 mm × 4.6 mm, 3.5 µm; 25% acetonitrile in 0.1 M ammonium formate (pH 3.5, prepared by mixing 1.1 mL of TFA with 1 L of 0.1 M ammonium formate); flow rate 1 mL/min, t*_R_* = ~6 min. 

Column (enantiomeric purity): Phenomenex, Lux ^®^ 5 µm Cellulose-1, 250 mm × 4.6 mm; 35% acetonitrile (0.1% acetic acid) in 10 mM ammonium formate (0.1% acetic acid); flow rate 1.5 mL/min, t*_R_* = ~7 min.

#### 3.1.3. Synthesis of [^18^F]SynVestT-1

A fully automated synthesis was performed using the Trasis AIO module. The reagent vials consist of (a) [^18^F]F^−^ in target water, (b) DMAPH·OTf (10 mg) in 1 mL dimethylacetamide (DMA, position 2), (c) anhydrous acetonitrile (position 8), (d) water (position 9), and (e) formulation buffer, 100 µL of 45 mM sodium phosphate (3 mM P/mL) in 3 mL of water (position 16). [^18^F]F^−^ in target water was passed through the PS-HCO_3_ cartridge (position 5) to retain [^18^F]fluoride. The cartridge was washed with anhydrous acetonitrile (position 8) and dried under a nitrogen atmosphere and under vacuum for 5 min. [^18^F]Fluoride from the PS-HCO_3_ cartridge was eluted with DMAPH.OTf in 1 mL DMA (position 2) into the reactor containing precursor **1** (2 mg, Scheme 1) and 9 mg of tetrakis(pyridine) copper(II) bis(trifluoromethanesulfonate) (Cu(OTf)_2_Py_4_). The reaction mixture was heated to 120 °C for 20 min, followed by cooling down to 50 °C. Water (5 mL, position 9) was added to the reactor. The reaction mixture was pulled into the 10 mL syringe (position 11) and transferred to the HPLC injection loop for purification using a semi-prep column. The purified product, [^18^F]SynVestT-1, was directly collected (~6 mL) in the product vial (position 15) and formulated with 100 µL of 45 mM sodium phosphate (3 mM P/mL) in 3 mL of water (position 16). In a representative radiolabeling, starting with 154 mCi (5.7 GBq) (Table 3), 39 mCi (1.4 GBq) of [^18^F]SynVestT-1 was in with a total volume of ~9 mL (calculated ~13% EtOH; GC indicated ~13%).

## 4. Conclusions

A fully automated, cassette-based production of [^18^F]SynVestT-1 has been developed without azeotropic drying of [^18^F]Fluoride using the Trasis AIO module. The cassette-based synthesis provides a standardized production platform, improving process robustness and reproducibility. The RCY was 30–37% with a high radiochemical purity (>98%) using 2 mg of precursor in 60 min. This approach will also be applicable for radiolabeling other PET probes via copper-mediated fluorination.

## Figures and Tables

**Table 1:** Key steps for the synthesis of [^18^F] SynVesT-1.

Literature Method [48]	Current Method
(i) Elution of fluorine-18 with K_2_CO_3_/KOTf from the QMA cartridge	(i) Elution of fluorine-18 with DMAPH.OTf from the PS-HCO_3_ cartridge
(ii) Azeotropic drying of [^18^F]fluoride	(ii) No need for azeotropic drying of [^18^F]fluoride
(iii) Copper-mediated fluorination (5 mg precursor)	(iii) Copper-mediated fluorination (2 mg precursor)
(iv) HPLC purification	(iv) HPLC purification
(v) Sep-Pak catch-and-release of the final product	(v) No need for Sep-Pak catch-and-release of final product
(vi) RCY, 20.6 ± 1.2%	(vi) RCY, 30–37%
(vii) Synthesis time, 80 min	(vii) Synthesis time, 60 min

**Table 2:** Effect of precursor, Cu(OTf)_2_(Py)_4_ and solvent on the radiochemical yield.

Entry	Amount of Cu(OTf)_2_Py_4_ (mg)	Solvent	Amount of 1 (mg)	^a^ RCY (%, Decay Corrected)
1	15	DMA	5	50
2			3	39
3			2	40
4			1	13
5	9	DMA	2	39
6	9		2	23 ^b^
7	6		2	21
8	3		2	13
9 ^c^	18		2	<2
10	9	DMF	2	11

Radiolabeling was performed with 60–100 mCi (2.2–3.7 GBq) of [^18^F]fluoride, 120 °C, 20 min. ^a^ Isolated RCY; ^b^ 110 °C, 20 min; ^c^ Literature condition: elution Cu(OTf)_2_Py_4_ (24 mg).

**Table 3:** A record of activities in various steps of the synthesis (Trasis AIO) for representative radiolabeling.

Steps	Time	Activity Sensors		
		PS-HCO_3_ mCi (GBq)	Reactor mCi (GBq)	Product Vial mCi (GBq)
Start of Synthesis	08:32	154 (5.7)	18 (0.7)	0
End of PS-HCO_3_ Drying	08:42	146 (5.4)	17 (0.6)	0
End of PS-HCO_3_ Elution	08:50	17 (0.6)	137 (5.1)	0
End of Radiolabeling	09:13	14 (0.5)	120 (4.4)	0
End of Synthesis	09:32	9 (0.3)	6 (0.2)	39.0 (1.4)

**Table 4:** Summary of three batches of [^18^F]SynVestT-1 production.

Entry	Batch 1	Bach 2	Bach 3
Starting [^18^F]F^-^	160 mCi (5.9 GBq)	145 mCi (5.4 GBq)	154 mCi (5.7 GBq)
[^18^F]SynVestT-1 (% yield, decay corrected)	33 mCi (1.2 GBq, 30%)	35 mCi (1.3 GBq, 35%)	39 mCi (1.4 GBq, 37%)
Appearance (visual inspection)	Colorless, clear, particle-free	Colorless, clear, particle-free	Colorless, clear, particle-free
Radionuclide identity, T_1/2_ (half-life)	109.5	108.5	109.5
Radiochemical purity (RCP)	>98%	>98%	>98%
Molar activity Ci/mmol (GBq/mmol)	14,800 (547,600)	15,700 (580,900)	16,400 (606,800)
Copper (ppm)	<0.1	<0.1	<0.1
Tin (ppm)	<0.1	<0.1	<0.1
Residual solvent, DMA (GC)	27 ppm	19 ppm	22 ppm
Chemical purity	>98%	>98%	>98%
Enantiomerical purity	>99%	>99%	>99%
Synthesis time (min)	60	60	60

## Data Availability

Data are contained within the article.

## Notes

This research was supported by the Intramural Research Program of the National Institutes of Health (NIH). The contributions of the NIH authors were made as part of their official duties as NIH federal employees, are in compliance with agency policy requirements, and are considered Works of the United States Government. However, the findings and conclusions presented in this paper are those of the authors and do not necessarily reflect the views of the NIH or the U.S. Department of Health and Human Services.
